# Effects of High-Forage Diets Containing Raw Flaxseeds or Soybean on In Vitro Ruminal Fermentation, Gas Emission, and Microbial Profile

**DOI:** 10.3390/microorganisms9112304

**Published:** 2021-11-05

**Authors:** Xiao-Ge Sun, Yue Wang, Tian Xie, Zhan-Tao Yang, Ji-Dong Wang, Yu-Hui Zheng, Cheng Guo, Yan Zhang, Qian-Qian Wang, Zhong-Han Wang, Wei Wang, Ya-Jing Wang, Hong-Jian Yang, Sheng-Li Li

**Affiliations:** 1 State Key Laboratory of Animal Nutrition, Beijing Engineering Technology Research Center of Raw Milk Quality and Safety Control, College of Animal Science and Technology, China Agricultural University, Beijing 100193, China; xiaogesun@163.com (X.-G.S.); S20203040614@cau.edu.cn (T.X.); yzt980929@163.com (Z.-T.Y.); sy20203040696@cau.edu.cn (J.-D.W.); zhengyuhui@cau.edu.cn (Y.-H.Z.); dxzxyan8@163.com (Y.Z.); qq_221w@163.com (Q.-Q.W.); wzh15675854231@163.com (Z.-H.W.); wei.wang@cau.edu.cn (W.W.); wangyajing2009@gmail.com (Y.-J.W.); yang_hongjian@cau.edu.cn (H.-J.Y.); 2 Animal Production Systems Group, Wageningen University & Research, 6708PB Wageningen, The Netherlands; yue3.wang@wur.nl; 3 School of Agriculture, Ningxia University, Yinchuan 750021, China; guochenghn@126.com

**Keywords:** oilseed, high-forage diets, volatile acid, CH_4_ emission, microbial communities

## Abstract

Lipid metabolism plays an important role in the energy economy of ruminants. However, its interactions of fat, rumen fermentation, gas emission, and microorganisms are not yet clear. This study evaluated the effect of adding raw oilseeds to high-forage diets on in vitro ruminal fermentation, gas composition, and microbial profile. Three isoenergetic and isoproteic experimental diets were designed and used as fermentation substrate: control treatment (CON group) was the basal diet lacking oilseeds, the other two treatments were the basal diet supplemented by 100 g/kg dry matter (DM) raw whole soybean (S group) and 50 g/kg DM raw flaxseed (F group), respectively. Data showed that the acetate, butyrate, and total VFA concentration of culture fluids in the S group were lower (*p* < 0.05) than in the F group. There was a tendency to a higher level (*p* = 0.094) of propionate concentration in the F group compared with the other two groups. The gas production in the F group was higher (*p* < 0.05) than in the control group. There was a lower abundance of *Sutterella* (*p* < 0.05) and a greater abundance of *Butyrivibrio* (*p* < 0.05) in both of the two oilseed treatments. *Methanobrevibacter* (*p* = 0.078) in the F group was the lowest. Our results suggested that CH_4_ emission could be inhibited with flaxseed supplementation by propionate production metabolism, biohydrogenation of unsaturated fatty acid (FA), and toxicity to *Methanobrevibacter*, while regarding soybean seed supplementation, the emission of CH_4_ was more likely to be reduced through biohydrogenation of unsaturated FA modulated by *Butyrivibrio*.

## 1. Introduction

Lipid supplementation in ruminant diets has been identified as an effective strategy to increase milk fat content and energy density of the ration [1]. Adding oil to ruminant feed can also manipulate the microbial profile and fermentation patterns in the rumen [2]. It was suggested that oils could positively affect rumen fatty acid (FA) proportions by influencing the activity of rumen microbes. Specific microbial communities and their interactions play an important role in many aspects of animal production, such as nitrogen cycles [3], methane (CH_4_) emissions [4], meat and milk quality [5]. Different sorts of fats may have different impacts on lipid metabolism in the rumen, the type of FA absorbed in the intestine and the final animal products. Dietary lipid has been studied from the perspectives of lipolysis, biohydrogenation pathways and microbial manipulate [6,7]. However, most of the research regarding polyunsaturated fatty acid (PUFA) has focused on milk production, milk profile of ruminants, and treatment of oil or processed (i.e., extrude, mechanical, protected) oilseeds [8,9]. The supplement of raw material oilseeds in the diet as well as its effects on the fermentation, CH_4_ emission, and microbes were rarely reported. It has been shown that both the amount and type of forage in the diet are important determinants of ruminal lipid metabolism [10]. Therefore, it is necessary to further assess the effects of adding specific fat sources to the high-forage diet on rumen fermentation, CH_4_ emission, and microbial profile.

The objective of our study was to determine the effect of raw whole soybean seed (enriched in n-6 FA) and flaxseed (enriched in n-3 FA) supplementation on ruminal fermentation, pH, gas emission, and microbial profile in vitro, when diets with high-forage (oat hay and corn silage) were incubated.

## 2. Materials and Methods

### 2.1. Diet Substrates

Three isoenergetic and isoproteic experimental diets were designed as fermentation substrates (Table 1). Control treatment (CON group) was the basal diet with forage supplying 70% of the dry matter (DM). Based on the diet in the CON group, the other two treatments were supplemented by raw whole soybean (S group) and raw flaxseed (F group), respectively. The DM contribution of raw whole soybean in the S group and that of raw flaxseed in the F group were 10% and 5%, respectively.

All samples were dried in an oven at 65 °C for 48 h until they had constant weights and were then ground in a small hammer mill to pass through a 1-mm sieve (40 mesh) before the determination of chemical compositions, according to the methods described in the AOAC [11].

### 2.2. Rumen Fluid Preparation

Three multiparous Holstein cows (daily milk yield of 25.3 ± 2.1 kg) with permanent rumen fistulas at mid-lactation period were used as rumen fluid donors. These cows had free access to water and were fed by a total mix ration (TMR) for ad libitum intake twice a day (6:00 a.m. and 6:00 p.m.). The ration consisted of 100 g alfalfa hay, 80 g alfalfa silage, 300 g corn silage, 100 g corn steam flake, and 420 g commercial concentrate per kg of DM. DM intake of the cows was 19.8 kg/d. The net energy of lactation and crude protein of the feed was 1.68 Mcal/kg and 17.3%, respectively. After 2 h of the morning feeding, rumen fluid was collected from each cow and strained into a plastic thermos through four layers of gauze (preheated at 39 °C). The collected rumen fluids from three donors were transferred into a beaker (capacity of 5000 mL), equally blended, and filled with CO_2_ at 39 °C in a preheated water bath.

### 2.3. Equipment and In Vitro Incubation

The cumulative gas production (GP) was recorded in real-time by the automated trace gas recording system (AGRS-III, Beijing, China) [13]. Buffer (pH 6.87) was configured according to Menke [14], and CO_2_ was continuously injected into the buffer for about 30 min prior to inoculation. For each incubation glass bottle (capacity of 120 mL), 0.5 g of substrates, 25 mL of filtered rumen fluids, and 50 mL of pre-heated buffer solution were added. All the bottles (3 treatments × 6 replicates × 5 time points = 90 bottles) were purged with N2 to make an anaerobic condition, rapidly sealed with butyl rubber stoppers and Hungate’s screw caps, and then immediately connected to the AGRS-III equipment by medical transfusion tubes. The samples were collected after being incubated at 39 °C for 3 h, 6 h, 12 h, 24 h, and 48 h, respectively. Meanwhile, an additional 18 bottles (3 treatments × 6 replicates = 18 bottles) prepared in the same manner were separately connected to the pre-emptied air bags and incubated at the same condition as above for 48 h for further analysis of gas composition.

### 2.4. Sampling

After the incubation, the bottles were disconnected from the AGRS-III system and air bag. Immediately, pH values of the culture contents were measured by sophisticated handheld pH meters (Starter 300; Ohaus Instruments Co. Ltd., Shanghai, China). All the fermented materials were separately filtered through pre-dried and weighed nylon bags (300 mesh, 9 cm × 14 cm) to obtain culture fluids. The nylon bags were firstly rinsed and clarified in tap water, then squeezed manually and dried in the oven at 65 °C for 48 h to determine the in vitro DM disappearance (IVDMD). The collected culture fluids were divided into five 2 mL sterile tubes. One of the aliquots was centrifuged at 4000× *g* for 15 min at 4 °C and the supernatants were collected. The supernatants were mixed with 0.2 mL of 250 g/L meta-phosphoric acid solutions at 4 °C for 30 min, then the mixture was centrifuged at 10,000× *g* at 4 °C for 10 min. Afterward, the supernatants were collected for the analysis of ammonium nitrogen (NH3-N) and volatile fatty acid (VFA). Another aliquot of culture fluids was stored at −20 °C for microbial proteins (MCP). The rest of the samples were stored at −80 °C, in which one aliquot was for further microbial community analysis and the rest were in reserve. The gas samples obtained from the air bag were used for gas profile analysis. Gas production techniques were based on the principle that anaerobic microbial digestion of carbohydrates releases gas (primarily CO_2_ and CH_4_) and VFA [15,16].

### 2.5. Chemical Analysis and Calculations

NH3-N and MCP concentrations were determined by spectrophotometry described in Verdouw et al. [17] and Cui et al. [18], respectively. The VFAs concentration and fermentation gas composition were measured with gas chromatography (6890 N; Agilent technologies, Avondale, PA, USA) proposed by Zhang and Yang [13] and Cui et al. [18], respectively.

IVDMD was calculated by the weight changes of substrates (DM basis) before and after in vitro incubation. The cumulative GP data were obtained by AGRS-Ⅲ system and fitted to a nonlinear model as the model (1) [19]:

GPt = A/(1 + (C/t)^B^) (1)

where GPt means the total gas production (mL/g DM) at time t (h), A means the asymptotic gas production (mL/g DM) at a constant fractional rate (c) per unit time, B is a parameter reflecting the shape of the curve, C means the time (h) at which reach the maximum gas production rate 1/2 and t mean the time of the gas recording.

Models (2)–(5) showed the calculations of the time at which the maximum rate of substrate degradation is reached (TRmaxS, h), the maximum rate of substrate digestion (RmaxS, h), the time at which RmaxG is reached (TRmaxG, h) and the maximum gas production rate (RmaxG, mL/h) [20]:

TRmaxS = C × (B − 1)^(1/B)^ (2)

RmaxS = (B × TRmaxS^(B−1)^)/(CB + TRmaxSB) (3)

TRmaxG = C × ((B − 1)/(B + 1))^(1/B)^ (4)

RmaxG = (A × CB × B × TRmaxG^(−B−1)^)/(1 + CB × TRmaxG^(−B)^)^2^ (5)

### 2.6. DNA Extraction and Determination

The ruminal fluid culture DNA was extracted using TIANGEN^®^ TIANamp Stool DNA Kit (Tiangen Biotech Co., Ltd., Beijing, China). DNA concentration and purity were determined using the NanoDropND-1000 spectrophotometer (NanoDrop Technologies, Wilmington, DE, USA). The V3-V4 region of the 16S rRNA gene was amplified according to the method described in Hao et al. [21]. As Guo et al. [22] reported, the former primer sequence of 341F (CCTACGGGNGGCWGCAG) and the reverse sequence of 806R (GGACTACHVGGGTATCTAAT) were used. Amplicons were extracted from 2% agarose gels, purified using the AxyPrepDNA Gel Extraction Kit (Axygen Biosciences, Union City, CA, USA), and quantified using the 7300 Real-Time PCR System (Applied Biosystems, Foster City, USA) with SYBR green chemistry (SuperReal PreMix Plus, Tiangen Biotech Co., Ltd., Beijing, China). Purified amplicons were pooled in equimolar mode and sequenced on an Illumina platform (Illumina Miseq PE300, Illumina, Inc., San Diego, CA, USA) to generate paired-end (2 × 250) sequences. The high-quality raw reads were outputted as FASTQ files and further selected according to FASTP (FASTQ preprocessor) (https://github.com/OpenGene/fastp accessed on 1 November 2022): reads with the unknown nucleotides (>10%) were removed. Paired-end clean tags were merged as raw tags by FLASH (version 1.2.11) shown in Magoč and Salzberg [23]. After merge, the sequences with a length less than 230 bp were removed by Vsearch (version 2.7.1). The raw tags were purified by the QIIME (version 1.9.1) pipeline under specific filtering conditions [24]. After eliminating noisy tags, high-quality tags were clustered into operational taxonomic units (OTUs) with ≥97% similarity using the Vsearch (version 2.7.1) according to the UPARSE method [25]. The representative sequences (highest abundance in each cluster) were classified into organisms by a naïve Bayesian model using the RDP classifier (version 2.2) [26], based on the SILVA 132 database (https://www.arb-silva.de/, accessed on 1 November 2022) [27], with confidence threshold values that ranged from 0.8 to 1. After annotation, species information can be obtained at genus level. If the sequence could not match with the annotation in the database, then it would not be annotated using other libraries. The unannotated sequences were classified as unidentified species. The abundance statistics of genus taxonomy were visualized with Krona (version 2.6) [28]. Alpha diversity indices (i.e., Chao 1, Observed species and Shannon) were calculated in QIIME (version 1.9.1). Sequence alignment by MUSCLE (version 3.8.31) [29] (http://www.drive5.com/muscle/, accessed on 1 November 2022).

### 2.7. Statistical Methods

The data of bacterial genes abundance, bacterial alpha diversity indexes (i.e., Chao 1, Observed species and Shannon), gas production, and composition were analyzed by one-way analysis of variance (ANOVA), and other data (i.e., IVDMD, pH, VFA, MCP, NH3) were analyzed by two-way ANOVA using SAS version 9.4 (SAS Institute Inc., Cary, NC, USA). Mean ± standard error of the mean (SEM) was shown, and effects significance was designated at *p* ≤ 0.05, whereas a tendency was assumed for 0.05 ≤ *p* ≤ 0.10. When the overall effect of the treatment was significant, mean comparisons were further tested using Bonferroni’s test.

## 3. Results

In the present study, the results showed that the raw oilseed treatments had different effects on the in vitro ruminal VFA, CH_4_ emission and microbial profile, while the IVDMD and pH were not affected.

### 3.1. In Vitro Dry Matter Digestibility

**Figure 1:**
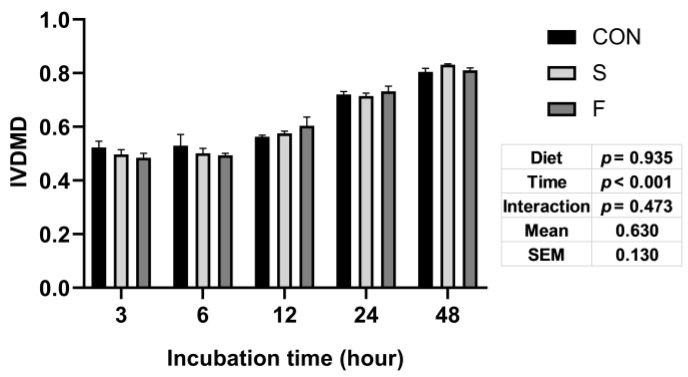
In vitro dry matter digestibility (IVDMD). The X-axis represents the incubation time (h). Y-axis represents the IVDMD value. CON: Control group, S: Raw whole soybean treatment, F: Raw flaxseed treatment. Diet: Diet treatment effects, Time: Incubation time effects, Interaction: The interaction effects between diet treatment and incubation time. Data are shown as the mean ± SEM, *n* = 6.

The overall means of IVDMD in these three diet treatments were not significantly different (Figure 1). The IVDMD increased along with the extended incubation time (*p* < 0.001). There were no interaction effects between diet treatment and incubation time (Figure 1).

### 3.2. Culture Fluids pH

**Figure 2:**
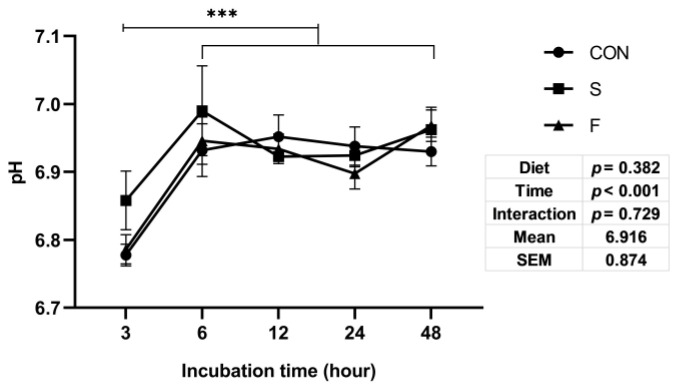
Effects of different oilseed treatments on in vitro pH. The X-axis represents the incubation time (hour). Y-axis represents the IVDMD value. CON: Control group, S: Raw whole soybean treatment, F: Raw flaxseed treatment. Diet: Diet treatment effects, Time: Incubation time effects, Interaction: The interaction effects between diet treatment and incubation time. ***: *p* < 0.01, means the culture fluids pH of incubation time 3 was significantly lower than other incubation times. Data are shown as the mean ± SEM, *n* = 6.

The culture fluids pH was not affected by the raw whole soybean and flaxseed addition (Figure 2). The pH in all groups had a significant increase (*p* < 0.01) from incubation time 3–6 and then kept at a relatively stable level (Figure 2).

### 3.3. Volatile Fatty Acid Concentration

As shown in Table 2, the acetate, butyrate, and total VFA concentration of culture fluids in the S group were lower (*p* < 0.05) than the F group, while both S and F groups were not significantly different from the CON group in those indicators. There was a tendency (*p* = 0.094) that the propionate concentration in the F group was higher than the other two groups. The acetate and butyrate ratio (A:P) was not affected by the diet treatment. Overall, the VFAs concentration of culture fluids increased (*p* < 0.05) along with the incubation time. The A:P elevated (*p* < 0.05) at incubation time 6 h and then went down (*p* < 0.05) (Table 2).

### 3.4. Ammonia–N Concentration

Compared with the CON group, S and F group tended to increase (*p* = 0.081) the NH3-N concentration of rumen culture fluids in vitro and accumulated over incubation time (Table 2).

### 3.5. Microbial Protein Concentration

The MCP concentration of culture fluids did not differ (*p* = 0.792) among the three treatments. It reached the highest concentration (721.01 µg/L) at incubation time 6 h and decreased to the lowest (549.03 μg/L) at incubation time 48 h (Table 2).

### 3.6. Gas Production and Kinetic Parameters

As shown in Table 3, the mean of GP_48_ and kinetic parameters in the F group was higher (*p* < 0.05) than the CON group, while there was no difference between S and CON groups. Other kinetic parameters (B, C, TRmaxG, RmaxG, TRmaxS, and RmaxS) were not affected by the supplementation of raw whole soybean and raw flaxseed (Table 3).

### 3.7. Gas Composition

**Figure 3:**
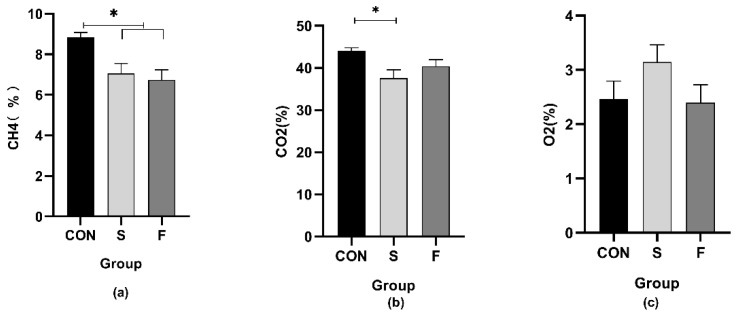
Effects of oilseed treatments on in vitro gas composition (%). (**a**): CH_4_ content, (**b**): CO_2_ content, (**c**): O_2_ content. CON: Control group, S: Diet treated with whole raw soybean, F: Diet treated with flaxseed. *: *p* < 0.05. Data are shown as the mean ± SEM, *n* = 6.

The CH_4_ proportion of the S and F groups were lower (*p* < 0.05) than the CON group, while there was no difference between S and F groups (Figure 3). The CO_2_ concentration of the S group was lower (*p* < 0.05) than the CON group, while no significant difference was found between those two groups and the F group (Figure 3). The O_2_ percentage was not shifted by any treatments (Figure 3).

### 3.8. Rumen Microbe Diversity and Abundance

**Figure 4:**
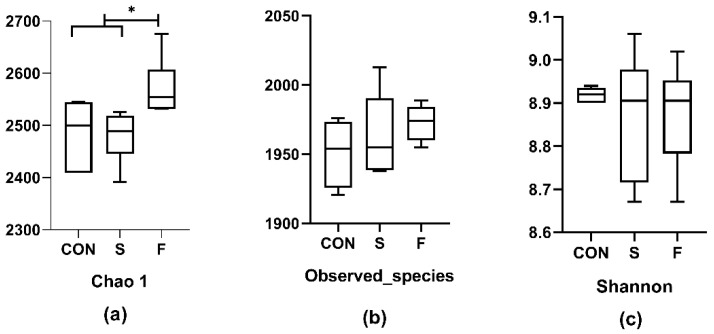
Alpha diversity indices for the sample bacterial communities. CON: Control group, S: Raw whole soybean treated group, F: Flaxseed treated group. (**a**): Richness of Chao 1 index, (**b**): Number of observed species, (**c**): Diversity estimator of Shannon. *: *p* < 0.05. Data are shown as the mean ± SEM, *n* = 6.

Chao 1 diversity index value for the F group was higher (*p* < 0.05) than the other two groups, while there was no difference between S and CON groups (Figure 4). The value of Observed species and Shannon did not differ among the three treatments (Figure 4).

As Table 4 shows, compared with the CON group, the relative abundance of *Sutterella* (*p* < 0.05) was lower in the S and F groups. In the S group, the *Hydrogenoanaerobacterium* (*p* < 0.05) was increased and the *Succiniclasticum* (*p* = 0.076) tended to increase. *Prevotellaceae_UCG-004* (*p* < 0.05) and *Prevotellaceae_Ga6A1* (*p* = 0.075) were highest in the F group, whereas *Methanobrevibacter* was lowest (*p* = 0.078). There was an increase in *Butyrivibrio* (*p* < 0.05) in the S and F groups compared with the CON group (Table 4).

## 4. Discussion

Unlike the study that showed fat supplementation might have negative effects on digestibility and fermentation parameters [30], this study found that rumen IVDMD and pH were not affected by the addition of oilseeds. It could be explained by the fat supplementation using whole oilseeds instead of vegetable oils has shown small negative effects on ruminal fermentation [8,31], and the high forage diet added with oil had little impact on rumen pH [32]. Compared with the CON group, the addition of flaxseed did not shift the concentration of most VFAs (i.e., acetate, butyrate, and total VFA) of rumen fluid in vitro, but increased the gas production and tended to increase the propionate concentration of rumen fluid. The results were in line with the study of Ueda et al. [32], which showed that flaxseed oil supplementation resulted in a greater molar proportion of propionate, but other VFAs were not affected. The acetate, butyrate, total VFA, and gas production per gram DM in the S group were lower than the F group. The results suggested that fat sources with a different FA profile may cause a reverse consequence. It could be partially explained by Jian et al. [33], which showed that alpha-linolenic acid (the main FA of flaxseed) could lead to significant higher total VFA than linoleic acid (the main FA of soybean). Additionally, the mean of GP_48_ and kinetic parameters in the F group were higher than the CON group, while there was no difference between the S and CON group. It implied that a diet with flaxseed added was more likely to enhance the fermentation parameters than that with raw whole soybean added.

In this study, dietary N and energy were formulated at the same level (Table 1). The NH3-N concentrations of rumen fluid with flaxseed and soybean supplementation tended to be higher than the CON group, but there was no difference in MCP among the three treatments. Previous studies regarding the effect of plant oil or oilseeds on NH3-N and MCP, however, had conflicting results. For instance, Pi et al. [34] showed that NH3-N and MCP were not affected by the addition of plant oil, whereas positive and negative effects were found in Scollan et al. [35] and Broudiscou et al. [36], respectively. Our results may be explained by the study of Ueda et al. [32], which showed ruminal NH3-N concentration was greater with the high-forage than with the high-concentrate diet, and was also greater with flaxseed oil supplementation, whereas the efficiency of bacterial N synthesis was not affected by either forage proportion or flaxseed supplementation. The NH3-N concentration significantly increased along with the incubation time. The MCP concentration was highest at incubation time 12 h and was lowest at incubation time 48 h. We took into account that the diet NH3-N was released to and accumulated in the rumen fluid culture, but the efficiency of bacterial N synthesis was decreased after an incubation time of 24 h.

The previous study showed that increasing the conserved forage (corn silage) in a roughage-based diet for dairy cattle can be an effective strategy to decrease enteric CH_4_ production [37], and diets supplemented with oilseeds could decrease CH_4_ output in ruminants fed conserved forages [38]. In our study, both the F and S group had lower CH_4_ emissions than the CON group. For the F group, it could be partially explained by enhanced propionate production, which was considered as a competitive pathway for hydrogen use in the rumen [39,40]. Moreover, the abundance of archaeal genes in ruminal digesta strongly correlated with methane emissions from individual animals [41], and Sousa et al. [42] found that lipids inhibited methanogenesis due to the toxicity of long-chain fatty acids to methanogenic archaea and *Methanobrevibacter* was one of the predominant genera of methanogenic archaea, which supported our results that the relative abundance of *Methanobrevibacter* tended to be declined in the F group. *Methanobrevibacter* are considered to be the predominant protozoa-associated methanogens [43]. However, during the treatment in the S group, the propionate production and *Methanobrevibacter* were stable, indicating that some other factors affected the CH_4_ emission. The *Butyrivibrio* was higher in S and F group when compared with the CON group, which was related to the biohydrogenation of unsaturated FA and might explain the reduction of CH_4_ [44]. Toral et al. [45] also suggested that *Succiniclasticum* played a part in the biohydrogenation processes. Accordingly, the S group had the highest abundance of *Succiniclasticum*, implying that the CH_4_ percentage was likely to be reduced by the biohydrogenation of unsaturated FA due to soybean seed addition.

At the genus level, except for the bacteria mentioned above, some other bacterial genes were affected by the treatments, including *Sutterella*, *Prevotellaceae_Ga6A1*, and *Prevotellaceae_UCG-004*. *Sutterella* was lower in the S and F group than the CON group, which was linked to the normal function of the colonic epithelium and inflammatory bowel disease [46]. *Hydrogenoanaerobacterium* was highest in the S group, and it belongs to the *Ruminococcaceae* family containing a large number of healthy gut-associated butyrate-producing bacteria [47]. Both *Prevotellaceae_Ga6A1* and *Prevotellaceae_UCG-004* were highest in the F group in this study. Bach et al. [48] reported that *Prevotellaceae_Ga6A1* was positively associated with feed efficiency. Meanwhile, the *Prevotellaceae_UCG-004* was highly connected with the efficiency of carbohydrate metabolism [49]. It suggested that adding soybean to the diet of ruminants could benefit the gut health, and the addition of flaxseed had the potential to increase the feed efficiency and carbohydrate metabolism.

## 5. Conclusions

In conclusion, this study showed the IVDMD, pH, VFAs, and the efficiency of bacterial N synthesis were not affected by the addition of oilseeds. However, the propionate, gas production, and kinetic parameters of the F group were significantly higher than the CON group. Overall, it implied that adding flaxseed to the diet was more likely to increase the fermentation parameters than adding raw whole soybean. Raw flaxseeds were able to reduce CH_4_ emissions through increased propionate formation, biohydrogenation of unsaturated FA and propionate toxicity to *Methanobrevibacter*, while raw soybean seeds tended to inhibit CH_4_ emissions through biohydrogenation of unsaturated FA modulated by *Butyrivibrio*.

## Figures and Tables

**Table 1:** Ingredients and chemical composition of the diets.

Ingredients	CON (DM %)	S (DM %)	F (DM %)
Oat hay	41.80	41.49	41.87
Corn silage	29.58	27.97	28.23
Corn fine	10.63	10.93	9.23
Soybean meal	16.99	8.61	14.67
Whole soybean	0.00	10.00	0.00
Flaxseed	0.00	0.00	5.00
Mineral vitamin premix	1.00	1.00	1.00
Total	100.00	100.00	100.00
Nutrition (DM basis)			
DM (kg)	12.42	12.51	12.4
CP (%)	13.85	13.77	13.78
NE_L_ (Mcal/kg)	1.48	1.49	1.49
NFC (%)	34.60	33.46	33.29
NDF (%)	42.00	41.95	42.41
EE (%)	2.60	4.01	4.01
UFA (%)	1.47	2.74	2.73
n-6 (g)	102.03	178.23	121.48
n-3 (g)	45.65	58.63	150.68
n-6:n-3	2.23:1	3.04:1	0.8:1

DM: Dry matter, CP: Crude protein, NEL: Net energy of lactation, a calculated value according to NRC [12], NFC: Non-fibrous carbohydrate, NDF: Neutral detergent fiber, EE: Ether extract, UFA: Unsaturated fat acid, n-6: C18:2 (Linoleic acid, LA), n-3: C18:3 (α- Linolenic acid, ALA). CON: Control treatment, S: Raw whole soybean treatment, F: Raw flaxseed treatment. Premix: 1 kg of premix included vitamin A 440,000 IU, vitamin D3 110,000 IU, vitamin E 4000 IU, niacin 400 mg, Ca 152 g, P 41 g, Cu 750 mg, Mn 1140 mg, Zn 2970 mg, I 30 mg, Se 36 mg.

**Table 2:** Effects of oilseed treatments on in vitro VFA concentration and N metabolism.

Item ^1^	Diet ^2^			Time ^3^					SEM ^4^	*p*-Value ^5^		
	CON	S	F	3 h	6 h	12 h	24 h	48 h		Diet	Time	INT
Acetate (mmol/L)	27.34 ^ab^	26.13 ^b^	27.67 ^a^	19.82 ^d^	24.67 ^c^	23.48 ^c^	30.17 ^b^	37.59 ^a^	6.67	0.024	<0.001	0.149
Propionate (mmol/L)	10.68	10.64	11.09	7.54 ^d^	9.19 ^c^	9.04 ^c^	12.95 ^b^	15.89 ^a^	3.16	0.094	<0.001	0.100
Butyrate (mmol/L)	4.21 ^ab^	4.13 ^b^	4.41 ^a^	2.72 ^d^	4.01 ^c^	3.96 ^c^	4.78 ^b^	6.01 ^a^	1.14	0.035	<0.001	0.087
A:P	2.54	2.50	2.53	2.63 ^b^	2.69 ^a^	2.59 ^c^	2.34 ^d^	2.35 ^d^	0.16	0.592	<0.001	0.973
TVFA (mmol/L)	44.12 ^ab^	42.28 ^b^	44.62 ^a^	30.88 ^d^	39.24 ^c^	37.85 ^c^	49.36 ^b^	62.03 ^a^	11.58	0.033	<0.001	0.118
NH3-N (mg/dL)	10.84	11.85	11.84	5.04 ^e^	6.89 ^d^	8.11 ^c^	13.84 ^b^	23.43 ^a^	6.93	0.081	<0.001	0.659
MCP (μg/L)	650.70	631.58	650.71	665.26 ^ab^	721.01 ^a^	644.74 ^b^	640.79 ^b^	549.03 ^c^	95.54	0.792	<0.001	0.660

^1^ A:P: Acetic acid: Propionic acid, TVFA: Total volatile fat acid, NH3-N: Ammonia–N, MCP: Microbial protein. ^2^ CON: Control group, S: Diet treated with whole raw soybean, F: Diet treated with flaxseed, ^a,b,c^: Different superscripts means significant differences in the indicators among groups (*p* < 0.05). ^3^ Time: Incubation time, ^a,b,c^: A row with different superscripts means they differ significantly under time effect factors (*p* < 0.05), *n* = 6. ^4^ SEM: Standard error of the mean. ^5^ Diet: Diet treatment effects. Time: Incubation time effects. INT: The interaction effects between diet treatment and incubation time.

**Table 3:** Effects of oilseed treatments on in vitro gas production and kinetic parameters.

Item ^1^	Diet ^2^			SEM ^3^	*p*-Value
	CON	S	F		
GP_48_ (mL/g)	67.48 ^b^	75.49 ^ab^	85.33 ^a^	13.26	0.032
A (mL)	69.36 ^b^	78.05 ^ab^	91.42 ^a^	15.97	0.024
B (h)	1.67	1.41	1.51	0.24	0.142
C (h)	6.48	7.09	7.69	1.23	0.194
TRmaxG (h)	2.61	2.38	1.92	0.69	0.157
RmaxG (h)	6.75	6.81	7.73	1.08	0.167
TRmaxS (h)	4.68	4.36	3.55	1.15	0.164
RmaxS (mL/h)	1.37	1.12	1.11	0.03	0.174

^1^ GP_48_: The cumulative gas production (mL/g DM) at incubation time 48 (h); A: The asymptotic gas production (mL/g DM); B: A sharpness parameter determining the shape of the curve; C: The time (h) at which half of A is reached and t is in vitro incubation time; TRmaxS: The time at which maximum rate of substrate degradation is reached (h); RmaxS: The maximum rate of substrate digestion (/h); TRmaxG: The time at which RmaxG is reached (h); RmaxG: The maximum gas production rate (mL/h). a,b: Different superscripts mean significant differences in the indicators among groups (*p* < 0.05), *n* = 6. ^2^ CON: Control group, S: Raw whole soybean treated group, F: Flaxseed treated group. ^3^ SEM: Standard error of the mean.

**Table 4:** Effect of different oilseed treatments on the relative abundance of bacterial genes.

Item	Diet (%)			SEM	*p*-Value
	CON	S	F		
*Sutterella*	0.173 ^a^	0.107 ^b^	0.123 ^b^	0.033	0.007
*Prevotellaceae_Ga6A1*	0.021	0.028	0.044	0.014	0.075
*Hydrogenoanaerobacterium*	0.006 ^b^	0.016 ^a^	0.011 ^ab^	0.005	0.029
*Butyrivibrio*	0.527 ^b^	0.678 ^a^	0.642 ^a^	0.078	0.015
*Succiniclasticum*	7.607	9.764	7.634	1.414	0.076
*Prevotellaceae_UCG-004*	0.219 ^a^	0.325 ^ab^	0.356 ^a^	0.076	0.038
*Methanobrevibacter*	0.021	0.027	0.006	0.015	0.078

CON: Control group, S: Raw whole soybean treated group, F: Flaxseed treated group. SEM: standard error of the mean. ^a,b^: Different superscripts mean significant differences in the indicators among groups (*p* < 0.05), *n* = 6.

## Data Availability

The data that support the findings of this study are available from the corresponding author upon reasonable request, and the sequencing data are available from NCBI. The BioProject number is PRJNA771210.
